# Construction and validation of a nursing care protocol in anesthesia^1^

**DOI:** 10.1590/1518-8345.2143.2952

**Published:** 2017-12-11

**Authors:** Cassiane de Santana Lemos, Vanessa de Brito Poveda, Aparecida de Cassia Giane Peniche

**Affiliations:** 2 MSc, Doctoral Student, Escola de Enfermagem, Universidade de São Paulo, São Paulo, SP, Brazil, Nurse, Surgery Center, Hospital Sirio Libanes, São Paulo, SP, Brazil.; 3 Post-doctoral degree, Professor, Escola de Enfermagem, Universidade de São Paulo, São Paulo, SP, Brazil.; 4 PhD, Professor, Escola de Enfermagem, Universidade de São Paulo, São Paulo, SP, Brazil

**Keywords:** Anesthesia, Nursing Care, Operating Room Nursing, Perioperative Nursing, Patient Safety, Checklist

## Abstract

**Objective:**

To construct and validate a nursing care protocol in anesthesia.

**Method:**

methodological study of face and content validation, judging clarity, relevance, pertinence and comprehensiveness of a care protocol, elaborated from the integrative review of previous literature and based on the conceptual model of assistance perioperative nursing of Castellanos and Jouclas. The protocol was evaluated by five anesthesiologists and nurses from the surgical center. The results were analyzed through the content validity index.

**Results:**

among the 119 items assessed by experts, 11 (9.2%) instrument items presented content validity index of <80% and were changed. The items with disagreement were related to the selection and availability of materials and equipment, especially before anesthetic induction. The content validity index, obtained for the different items, proposed after the amendments mentioned, ranged from 80 to 100%, in the three periods of anesthesia, indicating the proper validity of the proposed content.

**Conclusion:**

the nursing care protocol in anesthesia was considered valid.

## Introduction

Anesthesia allows the patient comfort during surgery, once the choice of the anesthesia category is based on the type, duration and approach required during the surgical procedure, associated with the clinical, mental and psychological conditions of the patient. Thus, the types of anesthesia are classified in general, regional and sedation^1^.

General anesthesia is necessary for procedures that require complete immobility and unconsciousness. It can be classified in three ways, defined as total venous, when only venous drugs are infused, such as propofol and etomidate, general inhalation, when administration of inhalational anesthetics like sevoflurane, desflurane. The association between venous and inhalational anesthetics is defined as general balanced anesthesia^1^ ^-^ ^2^.

The general anesthesia is constituted by the reversible unconsciousness, immobility, analgesia and autonomic reflex block, whose components are hypnosis, analgesia, muscle relaxation and neuro-vegetative block^2^.

Hypnosis is characterized by suppression of consciousness, obtained through the use of inducing agents such as midazolam, propofol and etomidate. Analgesia consists of the relief or absence of pain by the use of drugs such as opioids and anti-inflammatories. Muscle relaxation occurs through the reduction of muscle tone, with the administration of relaxants such as succinylcholine, atracurium and rocuronium. The neuro-vegetative block occurs after adequate hypnosis and analgesia, with an attenuated response of the autonomic nervous system to the surgical stimulus, such as changes in heart rate, blood pressure and sweating^2^.

Regional anesthesia is selected for surgical procedures that address upper or lower limbs, abdomen or pelvic region. It can be performed by epidural nerve block, subarachnoid or plexus nerve block, with the administration of local anesthetics such as xylocaine, ropivacaine^1^. The association between general and regional anesthesia is defined as combined anesthesia.

Sedation is aimed at patient comfort in small or ambulatory surgical procedures, contributing to the reduction of anxiety, analgesia, decreased movement and maintenance of hemodynamic stability, mainly respiratory pattern and cardiovascular function^1^ ^,^ ^3^.

Adverse events related to health care and patient safety endangerment originate from human error, lack of teamwork and organizational failures^4^. Better communication and collaboration among professionals may reduce the risk of morbidity associated with the care of the surgical patient^5^. In this context, health care planning, consisting of physicians, nurses and anesthesiologists, is essential to reduce the risks of morbidity and mortality during the anesthetic-surgical procedure, thus promoting patient safety.

Studies indicate that adverse events related to anesthesia involve mainly the respiratory system, cardiovascular system, errors in the execution of regional blocks, equipment and device failures, adverse reactions to medications and lesions related to surgical decision^6^ ^-^ ^7^.

The events of the respiratory system are caused by intubation difficulty, inadequate ventilation and oxygenation, aspiration, early extubation and airway obstruction^6^ ^-^ ^7^.

Concerning the cardiovascular system, adverse events are characterized by hemorrhages, bleeding, hydroelectrolytic imbalances and stroke. Equipment and device failures include the use of central and peripheral catheters, anesthesia equipment, burns and fires caused by the use of electric scalpel and thermal blanket^6^ ^-^ ^7^.

Adverse drug reactions are due to incorrect dose administration and inadequate analgesia^6^ ^-^ ^7^.

The main events in regional blocks are accidental dural puncture, high blockade, cardiac arrest, inadequate analgesia, trauma and catheter retention^6^ ^-^ ^7^.

It should be noted, however, that the mortality risk associated with the anesthetic-surgical procedure has been reduced in the last 50 years due to initiatives for patient safety in anesthesia, which produced a higher quality of perioperative care^8^ ^-^ ^9^.

These initiatives involved the advancement of training, certification and education of professionals for teamwork, development of medications and techniques, improvement of monitoring standards, greater quality in the risk assessment of the patient for surgery, along with the standardization behaviors through care protocols^8^ ^-^ ^9^.

For the same purpose of care, the health care security, the American Committee on Quality and Health Care of the Institute of Medicine (IOM) presented, in 1999, the report To err is human: building a safer health system, proposing the governmental creation of a patient safety center that defined national goals for promoting safety and preventing errors related to health care^10^.

As a result of mortality and morbidity rates caused by surgical procedures, in 2002, The National Assembly of World Health Organization (WHO), released a resolution aimed at safety during anesthetic-surgical interventions, establishing standards of quality health services, including the implementation of safe anesthesia^11^.

The Safe Surgeries Saves Lives program was launched in 2008 by the WHO, with the development of a checklist that directed the assistance performed before the anesthetic induction periods, surgical incision and removal of the operating room^11^ ^-^ ^12^.

The checklist, among several actions, provides for the anesthetic procedure, the checking of the functioning, availability of necessary equipment and materials, patient identification, verification of available tests, evaluation of risks associated with difficult airway via and blood loss^11^ ^-^ ^12^.

It has been shown in studies that the application of the checklist was recognized by health professionals as an important instrument for the prevention of errors, increase of patient safety and assurance of greater assertiveness in inter-professional communication, which enables the previous assessment of the risks and behavior decisions for damage prevention. Thus, there was a reduction of postoperative complications among patients undergoing elective and emergency surgeries, with the reduction in the reporting of adverse health events ^(^ ^13^ ^-^ ^16^.

Therefore, the care protocols, that is, the definition of a specific assistance/care situation, describing details about the operational actions and specifications about the mode of execution and professional execution, are instruments that can reduce the variability of conduct among the professionals involved in health care, to promote greater security for the patient and for the professional, to allow process and outcome indicators to be developed, to improve the quality of care and the rational use of resources^17^.

The behaviors recommended in the care protocols must be clear and accurate as to the expected results, to facilitate the use orientation and the understanding by the professionals^18^, besides being reviewed periodically, considering the local reality or application institution. 

The construction of protocols must be based on scientific evidence, according to their levels of recommendation, based on the elements of quality, quantity and consistency of the reviewed studies^18^.

The quality element corresponds to the set of evaluations of individual studies, considering the reduction of errors and bias. The quantity involves the number of studies, sample size or representative power. The consistency element is equivalent to the degree of similar results in different study designs^18^.

In Brazil, according to article 4º of Law 12,842 of July 10^th^, 2013, the execution of deep sedation, anesthetic blocks and general anesthesia are private activities of the physician^19^.

Regarding nursing, the world reality differs from the national one, with different levels of nursing autonomy in the anesthetic procedure. In the United States, there is a specialty of anesthesia in nursing, with certification^20^ and defined care standards, which allow the planning and execution of all anesthetic procedures by the nurse^21^.

The American nurse who holds the title of specialist in anesthesia conducts nursing undergraduate course and specialization courses of two to three years to obtain professional certification, which must be revalidated every two years^20^. The professional can act in a freelance/self-employed, or in anesthesia groups, composed of nurses and/or physicians.

The American nurse responsibilities include pre-anesthetic evaluation, application of a consent form for the procedure, preparation of the anesthesia plan in any type of procedure, insertion of invasive devices for hemodynamic monitoring, administration of drugs for induction and maintenance of anesthesia, control of the airways and ventilatory pattern during surgery^21^.

In some European countries, such as France, Bulgaria and Switzerland, the anesthetist nurse performs certification courses from one to three years, and can perform anesthesia, monitoring and insertion of invasive devices, through defined protocols and under the direct or indirect supervision of medical anesthesiologists^22^.

In Brazil, unlike other countries, nurses do not have specific legislation that allows them to act in anesthesia care, with the same autonomy already seen in the countries mentioned.

Thus, after the undergraduate course in nursing, the Brazilian nurse can choose the nursing specialization in surgical center, recovery room and sterilization central supply offered by different public and private educational institutions. It is necessary to emphasize that the absence of specialization does not prevent the Brazilian nurses from working in a surgical center. However, many health institutions require specialization courses and/or professionals with experience in perioperative care.

Therefore, the Brazilian surgical center nurse acts in the planning, management, execution of care and leadership of the nursing team^23^ ^-^ ^25^.

Care planning is performed by applying the nursing process, called the Perioperative Nursing Care System^26^. Among the nurse’s management activities is the control of equipment and materials required for the anesthetic-surgical procedure, management of the rooms for procedures, and supervision of activities and care performed by nursing technicians^24^ ^-^ ^25^.

The execution of the surgical patient care includes intraoperative monitoring, administration of drugs and blood components, bladder catheterization, assistance during anesthesia and surgical positioning, infection control through the availability of sterile materials and antisepsis measures^24^ ^-^ ^25^.

Regarding anesthesia care, the surgical center nurse acts in the planning of materials and equipment required, according to the type of anesthesia, patient monitoring, intubation monitoring and ventilation control during anesthetic induction, helps in the control of signs endotracheal aspiration and transport of the patient in the anesthesia reversal phase^27^.

However, there is no anesthesia care standard for Brazilian nursing professionals, so each institution performs a different practice and care varies according to the interaction between the anesthesiologist and the nursing team.

 The surgical center nurse encounters difficulties in the execution of the nursing process, due to the demand of the health institutions for the fulfillment of their assistance, administrative and managerial role. The difficulty is aggravated by the fact that health institutions do not understand the importance of the nurse’s role in assisting the surgical patient, leading to the misuse of assistance for managerial function^28^.

Therefore, in order for nurses in a surgical center to perform their role of care in a relevant way, it is necessary to master the scientific knowledge and specificities of the changes generated by anesthesia and surgery, for adequate care planning and evidence of the significant role in the health team.

Thus, in this study the objective was to construct and validate a nursing care protocol in anesthesia.

## Method

This is a methodological study of face validation and nursing care protocol content in anesthesia, based on results obtained in an earlier integrative review, evaluating the actions and care performed by the nursing team in the operating room during the anesthetic procedure^29^.

In order to construct the instrument, initially, the American and European anesthetist nurses standards^21^ ^,^ ^30^ ^-^ ^31^ were observed, considering the Brazilian nursing professional practice law^32^, as a basis for structuring the proposed interventions together with the care suggested by the WHO Safe Surgery Checklist^11^ ^-^ ^12^.

In accordance to the mentioned above, the theoretical framework that guided the construction of the care protocol was the conceptual model of perioperative nursing care, proposed by Castellanos and Jouclas^26^, which claims the care on the surgical patient in five stages: preoperative evaluation, identification of problems, care planning, implementation of nursing care in the intraoperative period and postoperative evaluation.

Considering the specificity of the proposed instrument, the stages proposed by the conceptual model of perioperative care were structured in three periods of anesthesia care: before induction (organize), induction of anesthesia (assist) and reversal (check).

The period before anesthetic induction consisted of interventions to organize the materials and equipment required for anesthesia. The period of anesthetic induction included direct assistance to the patient care and support to the anesthesiologist. In the reversal period, interventions were defined to check vital signs and record of the care performed.

The first version of the protocol was composed of interventions, involving the three assistance periods, evaluated by a group of five experts, regarding content validity criteria, clarity of items, relevance and completeness of content^33^.

The modifications of the interventions were performed regarding the presentation and structure, considering the result of the calculation of the Content Validity Index (CVI) for each intervention and suggestions of the experts.

The main changes suggested were the improvement of the writing of the items, especially in the pre-induction period, and separation of materials and equipment into distinct items, so that the professional only indicates the devices related to the type of surgery that will be performed^33^.

In the induction period, the judges suggested revisions of the items regarding nursing notes and records, avoiding duplication of information, checking the patient’s history and allergies directly in the medical chart, as well as reviewing the care of the anesthesiologist, such as assessment of ventilation and venous puncture^33^.

Before the suggestions, a second version of the care protocol was elaborated, with changes in the items regarding the appearance and form of presentation, followed by a new stage of face and content validation by five specialists.

In the second version of the care protocol, brief orientation of the objectives and form of completion of the instrument preceded the described periods. Following, the nursing interventions for each period were described.

The total validity of an instrument is measured by face, content, construct and criterion-related validity^34^. In this study, we sought to reach the first validation stages, that is, the structure and content of the protocol elaborated, through the evaluation by the judges of each item referring to its clarity, adequacy, relevance and completeness of content.

The validity of face represents how much a measure seems to be related to the specific content of the evaluated instrument, that is to say, if the content is understood by whom uses the instrument^34^. For the face evaluation, in this study, experts answered whether the graphic presentation, the orientation on the form of filling and readability, according to the sequence of presentation of the items, were adequate in the constructed protocol. 

Content validity consists of representativeness level of the concept that the instrument intends to measure and provides the items evaluation, according to clarity, relevance, pertinence and comprehensiveness of content^35^.

Clarity evaluates whether the construction of the items of the instrument, as the written form, allows adequate reading and promotes understanding of the content. The relevance indicates how the item represents the content that is being measured. Pertinence considers whether items of the instrument are suitable and specific to the assessment content. The completeness of content shows whether the instrument encompasses all items related you want to measure ^(^ ^35^.

Each item of the Protocol was scored from one to five, according to Likert type scale: (five) totally agree, (four) I agree, (three) neither agree nor disagree, (two) disagree, (one) totally disagree. On items where disagreement was found, the experts could suggest modifications regarding the proposed content.

The Likert scale is used to measure the opinions, beliefs or attitudes of the respondents of a questionnaire or instrument, through a sequence of affirmations that allows different levels of agreement. Depending on the research phenomenon and the researcher’s objectives, odd or pair number of response options may accompany each statement, and responses vary from totally disagree to totally agree^35^.

The researcher made e-mail contact with the experts and sent an electronic questionnaire (Google Docs), a presentation letter and the assistance protocol, defining the objectives of the study, instructions on the completion and importance of document evaluation by the experts.

In the scientific literature, it is recommended that the number of specialists selected varies between three and ten individuals^36^ ^-^ ^37^, five is considered to be suitable for evaluation of the agreement^38^.

The selection of experts may vary according to the time of clinical experience of the participants, publications and specialization in the area of the research phenomenon, so, in this study, the experts were selected for their work as researchers/specialists in the field of anesthesiology and/or perioperative nursing.

At the end of the protocol evaluation by the expert group, the data were treated and analyzed by the CVI, which measures the proportion or percentage of agreement among experts on certain items of an instrument^39^.

In this study, the highest scores were adopted for the CVI calculation, that is, responses (four) agree and (five) totally agree for each item, divided by the total number of specialists, excluding values from one to three. The acceptable agreement rate for this proportion was defined as 80% or higher^40^, with questions modification for those that did not reach this rate, according to the experts’ suggestions, and a new round of evaluation.

This study was approved by the Research Ethics Committee of the Nursing School of the University of São Paulo, under nº 612310.

## Results

The care protocol was evaluated by five specialists, four nurses and one anesthesiologist. Among the professionals, two are nursing professors and work in public education institutions, two nurses working at surgical center and one is a coordinator of private anesthesia service.

The age of the experts varied between 29 and 58 years (average of 41 years) and the average time of professional training was 17 years.

Regarding face validity, all experts stated that the graphical presentation, the guidelines for the filling form and the sequence structure of the items allowed an adequate reading and understanding of the care to be performed by the nurse during the anesthetic procedure.

Among the 119 items evaluated, only 11 (9.2%) items of the protocol presented CVI <80%, as presented in Table 1, and they were modified according to the experts’ suggestions. There were no items excluded.

Most of the disagreements among the specialists occurred in the period before anesthetic induction, in which the evaluated items presented CVI ranging from 40 to 100% in the clarity criteria, from 80 to 100% in relevance, from 60 to 100% in pertinence and from 40 to 100% in completeness of content.

Regarding the item of operation of the anesthesia equipment, the experts considered the need for guidance on the test of the equipment and the evaluation of its operation, taking into account the diversity of brands and models. In addition, experts recommended the establishment of a minimum number of available breathing circuit and modification of the wording of the item, definition of the saturation percentage (color modification) of soda lime and the need for exchange (Table 1).

**Table 1:** Content validity index of less than 80% of nursing care, in the period before induction and maintenance, according to evaluation criteria. São Paulo, SP, Brazil, 2016

Item / nursing care	Evaluation criteria			
	Clarity	Relevance	Pertinence	Completeness
**Before induction**				
***A) Operation of anesthesia equipment***				
**1) Perform anesthesia equipment test**	**40**	**100**	**100**	**60**
**2) To evaluate the availability of breathing circuit reserves according to the age range of the patient**	**80**	**80**	**80**	**60**
**3) Evaluate the color of soda lime**	**40**	**100**	**60**	**40**
**4) Blood pressure cuff**	**80**	**100**	**80**	**60**
**5) Multi-Parameter Monitor Alarm**	**60**	**100**	**100**	**60**
***D) Airway material***				
**6) Separate endotracheal tube according to the patient's age range, consider the availability of three different numbers**	**60**	**100**	**100**	**60**
***E) Organize materials for intubation***				
**7) Facial mask according to patient's age group**	**60**	**100**	**100**	**80**
**8) Nasogastric tube**	**80**	**100**	**80**	**60**
***F) Difficult airway***				
**9) Availability of tracheostomy cannula**	**80**	**80**	**80**	**60**
**10) Esophageal-tracheal tube(** ***combitube*** **) availability**	**100**	**80**	**80**	**60**
**Anesthetic induction**				
***C) Monitor the patient with***				
**11) Temperature**	**80**	**100**	**100**	**60**

The experts suggested the definition of the type of blood pressure cuff, according to the physical characteristics of the patient, together with the establishment of the Multiparameter monitor alarm, according to the patient’s age group (adult or child) (Table 1).

Regarding airway materials, there were considerations regarding the separation of the endotracheal tube of different sizes, according to the patient’s physical characteristics and type of surgery (Table 1).

The experts stated that for the clarity and completeness of content of the items describing the intubation materials, it is necessary to choose the patient’s face mask and gastric tube, considering characteristics such as age and weight (Table 1).

In the items on difficult airway, experts advised to better define the parameters of choice of materials such as the tracheostomy tube and the esophagus-tracheal tube (combitube) (Table 1).

In the period of anesthetic induction, the evaluated items presented CVI from 80 to 100% in the criteria of clarity, relevance and pertinence and CVI from 60 to 100% in the completeness of content. The only item with disagreement was patient monitoring, for which it was suggested to explain the type of sensor used for temperature verification (Table 1).

In the period of anesthesia reversal, there was no change in the initially proposed items, which presented CVI from 80 to 100% in the criteria of clarity and completeness of content, 100% in the criteria of relevance and pertinence.

Following the evaluation of the experts’ suggestions, the items were modified and submitted to a new round of evaluations and the CVI was recalculated, as presented in Table 2.

**Table 2:** Content validity index of nursing care, modified in the period before induction and anesthetic induction, according to evaluation criteria, in its final version. São Paulo, SP, Brazil, 2016

**Item / nursing care**	**Evaluation criteria**			
	**Clarity**	**Relevance**	**Pertinence**	**Completeness**
**Before induction**				
***A) Operation of anesthesia equipment*** 1) Perform the anesthesia equipment test according to the manufacturer's instructions, also verifying: connection of the equipment to the source of energy and battery, source of gases (oxygen, nitrous oxide, compressed air and vacuum) available, connected to the equipment and with pressure ≥50 Psi* or 3.2 kgf / cm ^**2†**^ **, full and closed vaporizers, no gas oscillation with closed flowmeters.**	**80**	**100**	**100**	**100**
**2) Provide a set of breathing circuit according to the patient's age range**	**100**	**100**	**100**	**100**
**3) Evaluate the saturation (color) of the soda lime and consider the exchange if more than 50% is violet**	**100**	**100**	**100**	**100**
**4) Blood pressure cuff, according to the patient's physical characteristics (weight, age)**	**100**	**100**	**100**	**100**
**5) Adjust multi-parameter monitor alarms, according to the age range of the patient**	**80**	**100**	**100**	**100**
***D) Airway material***				
**6) Separate endotracheal tube, according to the patient's physical characteristics (age, gender) and type of surgery, consider the availability of three different sizes**	**100**	**100**	**100**	**100**
***E) Organize materials for intubation***				
**7) air cushion mask, according to the physical characteristics of the patient (weight, age)**	**100**	**100**	**100**	**100**
**8) Gastric probe: children from 4 to 10 Fr** ^**‡**^ **/adults from 14 to 18 Fr** ^**‡**^	**100**	**100**	**100**	**100**
***F) Difficult airway***				
**9) Availability of tracheostomy cannula, according to the patient's physical characteristics (weight, age)**	**80**	**80**	**80**	**80**
**10) Availability of esophageal-tracheal tube (combitube), according to the patient's physical characteristics (height)**	**100**	**100**	**100**	**100**
**Anesthetic induction**				
***C) Monitor the patient with***				
**11) Thermometer**	**80**	**100**	**100**	**80**

**Figure 1:**
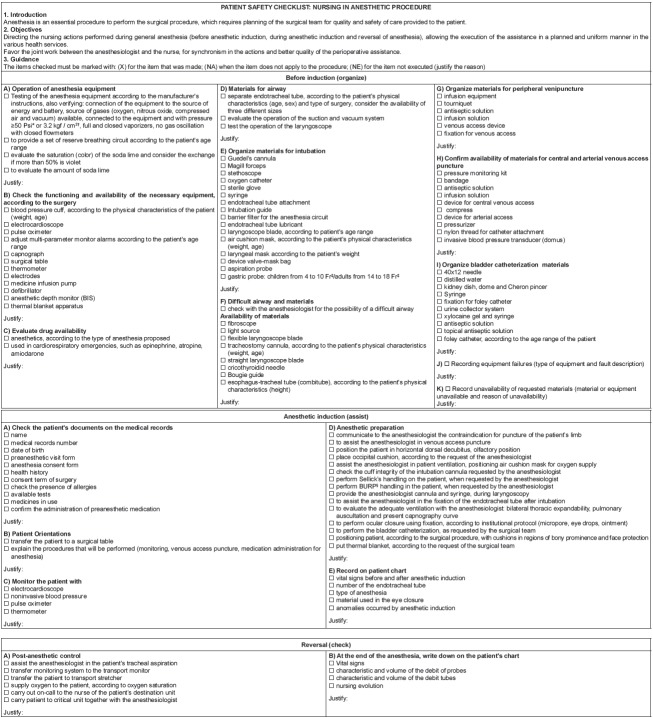
Final version of nursing care protocol in the anesthetic procedure. São Paulo, SP, Brazil, 2016

Figure 1 shows the final version of the care protocol.

*Psi: *pound force per square inch*; †kgf/cm^2^: *kilogram strength per square centimeter*; ‡Fr: *french*; §BURP: *backward, upward, right, pressure*

## Discussion

The results indicated a good agreement between the items evaluated by the specialists, considering that only 9.2% generated some type of disagreement and the divergences were mainly related to the criteria of choice, availability and selection of materials for the anesthetic procedure in the pre-induction period.

Most of the items of the induction period and all items of anesthesia reversal were considered valid by the specialists, affirming the importance of the care performed by the nurse in a surgical room.

The standardization of care offered by nursing during the anesthetic act contributes to the determination of the nursing role within this area, establishing a package of actions legally allowed in the country.

In Brazil, care and performance standards are clear for anesthesiologists^11^ ^-^ ^12^. The physician should perform the pre-anesthetic visit to evaluate patient risk factors, such as blood loss greater than 500 ml during surgery, assessment of the anatomy of the mouth and difficulty in intubation, according to Mallampati’s classification, risk of aspiration or airway allergies, alterations of tests, comorbidities and medications in use^11^ ^-^ ^12^.

During anesthesia-surgical procedure, the presence of an anesthesiologist is required in the surgical room, the supply of supplemental oxygen to patients undergoing general anesthesia, monitoring of oxygen saturation by pulse oximetry and ventilation, circulation control and heart rate monitoring, measurement of blood pressure every five minutes, body temperature measurement and regular evaluation of anesthetic depth by clinical observation^11^ ^-^ ^12^.

All these measures seek to prevent adverse events. Thus, it is considered that the definition of care standards, with the identification of potential risks and necessary measures for the safety of interventions, combined with inter-professional work and good communication, can improve health care processes and outcomes ^41^ ^-^ ^42^.

In 1993, the American Society of Anesthesiologists (ASA), aiming at the adequate planning of anesthesia care and reduction of postoperative morbidity and mortality, considering the reality of each health service and the types of equipment available, developed a checklist, revised in 2008, which contained actions for execution before anesthetic induction^43^.

The checklist suggested items, some of which are also covered by this protocol, such as the anesthesia equipment operating test, soda lime check, auxiliary oxygen supply availability and bag-valve-mask device, vacuum for aspiration, availability of monitoring system and alarms, with emphasis on pulse oximetry and capnography, verification of the flow of gases and adequate pressure for ventilation in the anesthesia equipment, as well as the registration of any conference procedure^43^.

It is worth noting that WHO recommends minimum standards of assistance/care in anesthesia during anesthesia-surgical procedures. They involve the planning of materials and medicines in health services, equipment testing, monitoring care, according to the complexity of the clientele served and the hospital institution. These measures can be defined as highly recommended, recommended and suggested^11^ ^-^ ^12^.

The highly recommended standards in anesthesia are mandatory standards, that is, applicable to all types of institutions that perform procedures under anesthesia, from small hospitals to referral hospitals.

Therefore, several items contemplated in the protocol must be properly checked and recorded, among them the availability and operation of materials in the operating room and materials for airway (masks, working laryngoscopes, endotracheal tubes, intubation guide (Bougie), oral and nasopharyngeal Guedel cannula, aspiration system and humidifiers), evaluation of anesthesia equipment (gas flow and pressure, soda lime color check, circular system with test balloons, full and closed oxygen cylinder, filled and adjusted vaporizers), (Lignocaine, diazepam, midazolam, morphine, adrenaline, atropine, inhaled anesthetics), blood and fluids available, monitors with alarms attached, stethoscope, sphygmomanometer and thermometer^11^ ^-^ ^12^.

Studies have shown that the use of checklists for the organization of anesthetic induction care can assist in the detection of errors and negligence in relation to care, reducing failures during anesthesia and surgery, by means of appropriate equipment and material checks, in addition to promoting the exchange of information among professionals about the clinical conditions of the patient and critical aspects such as allergies and difficult airway, thus improving the perception of professionals about teamwork and prevention of damages, aspects that, together, contribute for patient safety^44^ ^-^ ^46^.

All the professionals aligned with the scientific basis that guides the care to be performed, as well as the definition of limits and performance roles, contribute to a greater synchronism between the anesthetic procedure and the activities, and, therefore, a greater probability of success interventions. As a limitation, in this study, no pilot-application of the protocol was performed.

## Conclusion

The nursing care protocol in anesthesia was validated with good agreement among specialists, and 90.8% of the items were considered adequate in the first round.

The nursing care protocol was developed within the limits of professional performance, without overlapping functions or violation of the law of professional practice, and can guide the role of the nurse during anesthesia. With this, it is possible to standardize care behaviors in different institutions, guaranteeing greater support to nurses in their performance in the anesthetic procedure, with the anesthesiologist.

It is emphasized that the nurse’s use of the care protocol should be linked to the existence of systematized technical and scientific knowledge about the actions to be performed during anesthesia, stating the importance of their presence in a surgical room for care activities.

Thus, it is believed that this direction of nursing work, in anesthesia, can evidence the importance of nurses’ work in a surgical center, even in the face of the difficulties experienced in the performance of roles within health institutions and in interaction with the medical team.

Future work is needed to evaluate the practical application and feasibility of using the care protocol in different realities.
